# Identification and florfenicol-treatment of *pseudomonas putida* infection in gilthead seabream (*Sparus aurata*) fed on tilapia-trash-feed

**DOI:** 10.1186/s12917-024-04004-z

**Published:** 2024-04-25

**Authors:** Ibrahim M. Aboyadak, Mohsen Abdel-Tawwab, Nadia G. Ali

**Affiliations:** 1 National Institute of Oceanography and Fisheries, NIOF, Cairo, Egypt; 2 Department of Fish Biology and Ecology, Central Laboratory for Aquaculture Research, Agricultural Research Center, Abbassa, Abo-Hammad, Sharqia 44662 Egypt

**Keywords:** *Sparus aurata*, Trash fish, *Pseudomonas putida*, Pathogenicity, Florfenicol treatment

## Abstract

The present study aimed to determine the major cause of the high mortality affecting farmed gilthead seabream (*Sparus aurata*) and controlling this disease condition. Fifteen diseased *S. aurata* were sampled from a private fish farm located at Eldeba Triangle, Damietta, fish showed external skin hemorrhages, and ulceration. Bacterial isolates retrieved from the diseased fish were identified biochemically as *Pseudomonas putida* and then confirmed by phylogenetic analysis of the 16 S rRNA gene sequence. *P. putida* was also isolated from three batches of tilapia-trash feed given to *S. aurata*. Biofilm and hemolytic assay indicated that all *P*. *putida* isolates produced biofilm, but 61.11% can haemolyse red blood cells. Based on the antibiotic susceptibility test results, *P. putida* was sensitive to florfenicol with minimum inhibitory concentrations ranging between 0.25 and 1.0 µg mL^− 1^, but all isolates were resistant to ampicillin and sulfamethoxazole-trimethoprim. Pathogenicity test revealed that *P. putida* isolate (recovered from the tilapia-trash feed) was virulent for *S. aurata* with LD_50_ equal to 4.67 × 10^7^ colony forming unit (CFU) fish^− 1^. After intraperitoneal (IP) challenge, fish treated with 10 mg kg^− 1^ of florfenicol showed 16.7% mortality, while no mortality was recorded for the fish group that received 20 mg kg^− 1^. The non-treated fish group showed 46.7% mortality after bacterial challenge. HPLC analysis of serum florfenicol levels reached 1.07 and 2.52 µg mL^− 1^ at the 5th -day post-drug administration in the fish groups received 10 and 20 mg kg^− 1^, respectively. In conclusion, *P. putida* was responsible for the high mortality affecting cultured *S. aurata*, in-feed administration of florfenicol (20 mg kg^− 1^) effectively protected the challenged fish.

**Supplementary Information:**

The online version contains supplementary material available at 10.1186/s12917-024-04004-z.

## Introduction

Aquaculture is one of the fastest-growing animal production sector worldwide [1]. The global aquaculture finfish production reached 87.5 million tons in 2020 [2]. Nowadays, the expansion in aquaculture is an urgent need, especially with the current complex economic situation of the world, accompanied by increased food prices [3]. Aquaculture represents the golden solution for this problem as fish protein is much lower in price than red meat and poultry; however, fish had the lowest feed conversion ratio among other farm animals. Aquaculture is also the most sustainable and eco-friendly animal protein source; each 10 kg of cultured fish produces the same CO_2_ amount required for producing only one kg of red meat [4].

Gilthead seabream (*Sparus aurata*) is one of the most cultivated marine fish species in the Mediterranean region; and it occupies the thirty-three level among cultured fish species at the global scale [5]; world production reached 258,754 tons in 2019. Turkey, Greece, and Egypt are the top three producers; they produced 38.54%, 21.43%, and 13.87% of its total global production [6]. *S. aurata* was the first cultured marine fish species in Egypt; commercial production started in 1976 [7], the Egyptian production reached 35,880 tons in 2019 [8].

Bacterial diseases are the major challenge facing cultured freshwater and marine fish species resulting in massive loss in fish productivity [9]. Many bacterial pathogens were isolated from cultured seabream [10–15]. Among these pathogens, the genus *Pseudomonas*, a Gram-negative Gammaproteobacteria, was first described in 1894 and it comprises more than 191 described species [16]. *Pseudomonas* species are widely distributed in the aquatic environment and can colonize many niches like water and soil of fishponds. *P. putida* was isolated from diseased rainbow trout (*Oncorhynchus mykiss*), *Oreochromis niloticus*, European sea bass (*Dicentracchus labrax*), and *Liza ramada* [17–21].

Antibiotics are generally used to control and treat the bacterial infection in fish farms. Florfenicol is a broad-spectrum bacteriostatic fluorinated synthetic analog of thiamphenicol [22]. It is active against many bacterial pathogens by binding to the peptidyl transferase site at the 70 S sub-ribosomal unit and inhibits bacterial protein biosynthesis [23]. Florfenicol was approved for use in aquaculture by the Food and Drug Administration Organization (FDA) in 2005 and is currently one of the most extensively used antibiotics for treating bacterial fish diseases [24].

The present study aimed to isolate and identify the pathogenic *P. putida* responsible for the mass mortality in the studied gilthead seabream (*S. aurata*). Additionally, this study investigated the suitability of florfenicol as an effective treatment for *S. aurata* against *P. putida* infection.

## Materials and methods

### Fish sampling and clinical examination

Clinically diseased gilthead seabream (*S. aurata*) samples were collected alive from a local fish farm at Eldeba Triangle, Damietta, Egypt. This farm used tilapia as trash feed to feed the cultured gilthead seabream. Freshly dead and moribund *S. aurata* fingerlings were inspected for any external disease signs, and fish were dissected to record any internal lesions according to the method described by Austin and Austin [25].

Fifteen clinically diseased *S. aurata* were collected alive; fish samples were placed separately in a sterile plastic bag. Three trash feed (tilapia) samples were collected, each representing a separate feed patch. Each feed sample contained 100 g of chopped tilapia. Diseased fish and tilapia-trash feed samples were stored in the car refrigerator (− 8 °C) and then immediately transported to the laboratory as mentioned by El-Bahar et al. [26].

### Bacteriological assay

Swaps from the liver and the posterior kidney of each diseased *S. aurata* fingerlings were added to the tryptic soy broth, Oxoid, UK, TSB tubes were incubated at 37 °C for 24 h. Marine agar plates were streaked from the corresponding broth tube and incubated at 37 °C for 48 h.

The recovered bacterial isolates were identified by the Vitek 2 Compact system, bioMérieux, France, as described by Ali et al. [27] using the Gram-negative bacterial identification card. The biochemical profile of each isolate was automatically monitored and then reported by the system.

### Isolation of *P. putida* from tilapia-trash feed

Three trash fish (tilapia) patches were screened for *P. putida*. Briefly, 20 g of tilapia was homogenized in 100 mL peptone water at 10,000 x g for 10 min, and 10 mL from the homogenate was centrifuged at 2000 x g for 2 min. Five-hundred µL from the supernatant was added to 5 mL of *Pseudomonas* selective broth, HiMedia, India, supplemented with Cetrimide - Nalidixic acid (CN) supplement, and then broth tubes were incubated at 37 °C for 48 h. *Pseudomonas* selective agar plates with (CN) supplement (HiMedia), India were streaked from the broth tube and then were incubated at 37 °C for 48 h. The recovered colonies were biochemically identified by Vitek 2 Compact system.

### Molecular identification

#### Genomic DNA extraction

Bacterial genomic DNA was extracted using a G-spin™ total DNA extraction kit, Intron, Korea, as Ali et al. [28] described with few modifications. Briefly, 24-hour Tryptic soy broth (TSB) was centrifuged at 6000 x g for 10 min, 200 µL of CL buffer was added to the bacterial pellet, and then the tube was vortexed for one minute, followed by the addition of 20 µL of Proteinase K and 5 µl of RNase. Tubes were incubated at 56 °C for 30 min till complete lyses.

#### Amplification of 16 S rRNA gene

The 16 S rRNA gene amplification was performed according to Fujiyoshi et al. [29] using the universal bacterial primers 27 F (5′-AGAGTTTGATCCTGGCTCAG-3’) and 1492R (5′-GGTTACCTTGTTACGACTT-3′), Intron, Korea. Polymerase Chain Reaction (PCR) reaction mixture consists of 1.25 µL of each primer, three µl of genomic DNA, 12.5 µl of 2X master mix (i-StarMAXTM II, Intron), and 7.0 µL of nuclease-free water. Each cycle started with the initial denaturation at 98 ºC for 2 min, followed by 35 cycles of denaturation at 95 °C for 1.0 min, annealing at 60 ◦C for 1.0 min, and elongation at 68 °C for 3.0 min; finally, PCR run was ended with a final extension step at 72 °C for 10 min. The amplicon was detected by electrophoresis of PCR products in 1.5% w/v agarose gel supplemented with 0.5 µg/ml ethidium bromide, as mentioned by Lee et al. [30].

#### Sequencing

Twenty-five microliters of PCR product were purified using the MEGA quick spin™ total fragment DNA purification kit, Intron, according to the manufacturer’s instructions. Automated DNA sequencer system 3130, Applied Biosystems, USA, was used for forward sequencing of the purified PCR product.

#### Phylogenetic analysis

Phylogenetic analysis was performed for two bacterial isolate sequences (one from infected *S. aurata* “KT 2440” and the other from the trash fish used for fish feeding “PP”). Basic Local Alignment Search Tool (BLAST®) software was used to determine the obtained sequence identity to the GenBank data [31]. MEGA 9 program was used to draw the optimal phylogenetic tree using the neighbors-joining method [32].

#### Hemolytic assay

The hemolytic activity of *P. putida* products was assayed against *S. aurata* red blood cells (RBCs) according to the method described by Evans et al. [33] with some modifications. The overnight broth culture of *P. putida* was filtrated with a sterile syringe filter (0.45 μm) and then re-filtered with a polytetrafluoroethylene (PTFE) syringe filter (0.22 μm). One mL of blood was collected from the caudal vessels of healthy *S*. *aurata*; blood was centrifugated at 5000 x g for 5 min. The sedimented RBCs were washed three times with sterile 8.8% sodium chloride (pH:7.4) and then diluted as 1 RBCs to 50 phosphate buffers saline V/V. Ten µL from the culture filtrate was added to 190 µL diluted RBCs in a microtiter plate (in triplicates), followed by incubation at 27 °C for 12 h. The appearance of sedimented RBCs as a button shape with clear supernatant indicated a negative result.

#### Biofilm production assay

All the recovered *P. putida* isolates were assayed for biofilm production using the crystal violet colorimetric assay method described by Corte et al. [34]. Overnight bacterial growth on TSB was harvested by centrifugation. Bacterial cells were washed twice with sterile phosphate buffer saline and resuspended, then adjusted to 1.0 *×* 10^6^ CFU mL ^− 1^. In a microtiter plate, 25 µL from each isolate was loaded in three successive wells, and then 175 µL from sterile tryptic soy broth was added. The plate surface was covered with an adhesive tab and then incubated at 27 °C for 24 h. The broth was carefully removed from each well using a multichannel pipette and then washed twice with PBS. The microtiter plate was dried for 15 min in the incubator, immersed in 1% crystal violet for 15 min, washed with distilled water twice, and loaded with absolute methanol. The development of a violet color indicated a positive result.

### Antibiogram tests

#### Agar disc diffusion

The agar disk diffusion test was performed for all the recovered *P*. *putida* isolates (*n* = 18) according to the method described by Ali et al. [35]. Susceptibility for florfenicol (10 µg), ampicillin (10 µg), erythromycin (15 µg), sulfamethoxazole-trimethoprim (25 µg), ciprofloxacin (5 µg), doxycycline (30 µg), and tylosin (15 µg), Oxoid, UK. Muller-Hinton agar plates (Oxoid, Uk) were incubated at 37 °C for 48 h due to the slow growth rate of the tested isolates. The inhibition zone (mm) was measured to the nearest mm and interpreted according to the breakpoints mentioned [36].

#### Broth dilution test (minimum inhibitory concentration)

MIC for florfenicol and ciprofloxacin was determined, as described by Ali et al. [37] where 12.8 mg of antibiotic was added to 1.0 mL of distilled water for preparing the antibiotic-standard solution (1280 µg/100 µL). Double-fold serial dilution was performed for 15 successive dilutions. *P. putida* overnight cultured broth was adjusted to 0.5 McFarland standard and then diluted to 0.5% with sterile broth. Afterward, tetrazolium chloride was added to achieve a final concentration of 0.0001%. Precisely, 4.9 mL from the seeded broth was loaded into each sterile screw-capped tube, followed by the addition of 100 µL from the prepared antibiotic solution to the corresponding test tubes in final concentrations of 265, 128, 64, 32, 16, 8, 4, 2, 1, 0.5, 0.25, 0.125, 0.0625, 0.03125, and 0.015625 µg mL^− 1^, respectively. The last tube was left antibiotic-free as a negative control. Tubes were incubated at 37 °C for 24 h. The MIC is the lowest antibiotic concentration that stopped bacterial growth and preserved the broth color unchanged, and red-colored broth indicated bacterial growth.

#### Experimental fish and the infectivity test

Healthy *S. aurata* fingerlings were transported to the wet laboratory, NIOF, Alexandria, Egypt, under the optimum condition, as mentioned by Fang et al. [38]; continuous aeration was maintained during transportation using pure oxygenation. In the wet laboratory, fish were observed for 15 days during the acclimatization period. Five fishes were randomly selected and dissected for bacterial isolation using the same procedure used for diseased fish and were completely free from any bacterial infections.

The infectivity test for *P. putida* isolated from tilapia-trash-feed was performed according to Saleh et al. [39] to study the ability to induce disease in healthy fish. A single bacterial colony was picked up from the *Pseudomonas* selective agar plate and then incubated on brain heart infusion broth at 37 °C for 12 h. Bacterial growth was harvested from the broth by centrifugation, the optical density was adjusted to second McFarland standard then and then diluted to 5 × 10^6^, 5 × 10^7^, and 5 × 10^8^ CFU mL^− 1^ as described by Ali et al. [35]. Gilthead seabream fingerlings (21–25 g) were randomly divided into four equal groups in triplicate aquaria (each replicate aquarium contained ten fish). Each replicate was maintained in a separate glass aquarium (100 L) containing seawater at 24 ± 1 °C, tricaine methane sulfonate (Syncaine®), Syndel, USA was used as fish anesthetic at a dose of 25 mg.L^− 1^. Fish in each group were intraperitoneally injected (IP) with bacterial doses of 0.0 (control), 5 × 10^6^, 5 × 10^7^, and 5 × 10^8^ CFU mL^− 1^ [35]. Afterward, fish in experimental groups were observed twice daily for seven days to determine the fish mortality rate; dead fish was considered only after the re-isolation of *P. putida*, and the lethal dose (LD_50_) was calculated as described by Reed and Munch [40].

After the end of the study, the remaining fish were euthanized using Tricaine methane sulfonate (Syncaine®), Syndel, USA at a dose of 500 mg L^− 1^. Fish left in the anesthetic solution for 5 min after complete cessation of opercular movement then left in the refrigerator for 2-hours then burned.

During the fish acclimation period and the experimental running, physico-chemical water characteristics were regularly monitored. Water temperature and dissolved oxygen (YSI Professional Series Instrument, USA), pH values (pH/temperature branch meter, Italy), and salinity (Portable Refractometer, Operating Instructions, GG-201/211) were measured in site. Total ammonia-nitrogen (TAN), unionized ammonia (UN), and nitrite were assayed according to Boyd [41]. The water temperature was 29.6 ± 0.4 ◦C, dissolved oxygen was 5.18 ± 0.28 mg/L, the pH degree was 8.42 ± 0.28, salinity level was 25 ± 1 g/L, TAN was 0.31, UA was 0.053, and nitrite was 0.001; These parameters are within the acceptable range suitable for fish culture [42].

#### The florfenicol-fish treatment trial

The treatment trial was carried out to determine the efficacy of florfenicol (Nuflor, MERK, USA) in protecting *S. aurata* fingerlings against *P*. *putida* infection. Acclimated *S. aurata* fingerlings (21–25 g) were randomly divided into four groups with three replicates per each (10 fish/100-L aquarium). Fish in the first and the second groups received florfenicol-medicated chopped tilapia at a dose of 10 and 20 mg kg^− 1^ treated fish biomass, respectively. Fish in the third and fourth groups received non-medicated chopped tilapia. Florfenicol was added and thoroughly mixed with chopped tilapia 30 min before *S. aurata* feeding; the drug dose calculation was based on (3%) daily feeding rate of biomass. Fish in all groups were IP infected with *P*. *putida* (6.3 × 10^7^ CFU fish ^− 1^), while fish in the fourth group were IP injected with saline solution and kept as a negative control. Blood samples were collected from the caudal vessels (6 fish/group) at 24-h post-drug administration and then daily to avoid non-specific mortality that may result from blood sampling.

### HPLC determination of florfenicol in treated fish serum

#### Blood samples

Blood samples were collected from the caudal vessels one hour before experimental infection (24 h after the first and 24 h after the last dose). Serum samples were separated by centrifugation at 2000 x g for 5 min, then preserved at − 18 °C until analysis.

#### Samples preparation for analysis

Serum samples were deproteinized using 60% perchloric acid to serum (1:20 V/V). Deproteinized serum samples were centrifuged at 6000 x g for 10 min, and then the supernatant was filtered with 0.22 μm PTFE syringe filter.

#### Chromatographic reagents and conditions

Florfenicol analytical standard (99.7% Sigma-Aldrich, HPLC grade methanol), bi-distilled water, and acetonitrile were used for preparing the standard solution and mobile phase. Florfenicol concentration was assayed in Nexera X2 HPLC system, Shimadzu, Japan, equipped with UV detector and Zorbax C18 column, Agilent, USA, (4.6 mm internal diameter × 250 mm length and 5 μm particle size). The assay method was performed as described by Yang et al. [43] at 225 nm using acetonitrile-water (35:65 v/v) as mobile phase at 0.6 mL min-1 flow rate and 50 µL inoculum volume.

#### Statistical analysis

The data obtained in fish experiments were tested for distribution normality and variance homogeneity according to Kolmogorov-Smirnov and Bartlett’s tests, respectively. Data of fish mortality after bacterial IP infection doses were subjected to one-way ANOVA. On the other hand, two-way ANOVA was used to determine the effects of florfenicol application dose and sampling time affected florfenicol residues in fish sera. Tukey’s HSD was used as a post-hoc test to confirm the differences among means at *P* < 0.5. All statistical analyses were performed using the SPSS software version 26 (SPSS, Richmond, VA, USA) as described in Dytham [44].

## Results

### Clinical inspection

**Fig. 1:**
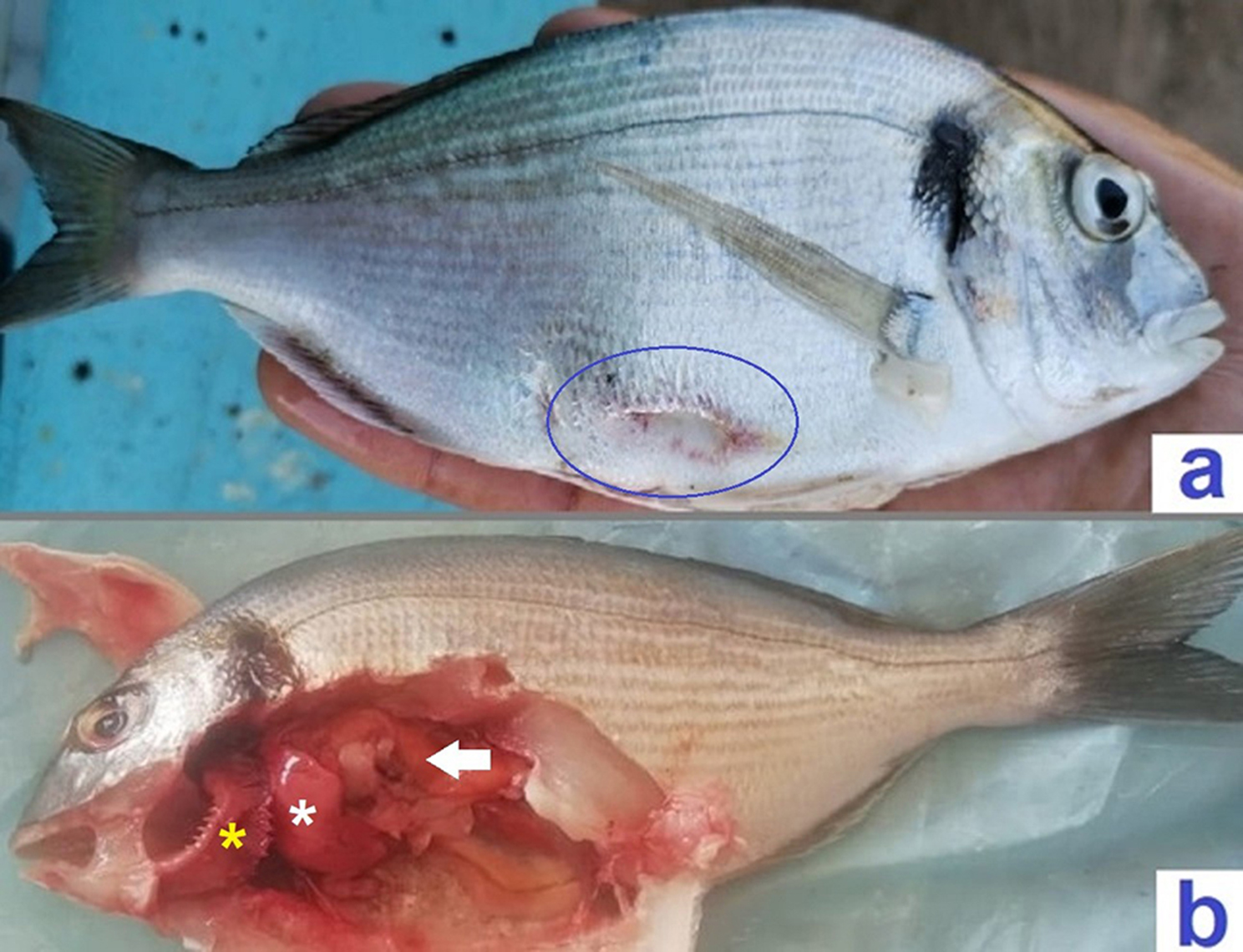
(**a**) Skin ulcers surrounded with hyperemia (blue circle) in naturally infected *Sparus aurata*. (**b**) Congested liver with the presence of petechial hemorrhage (white Asterix), congested partially empty stomach (white arrow) and severely congested gills (yellow Asterix)

Skin hemorrhages are the most prominent clinical sign appeared on the diseased fish. The liver and posterior kidney were congested in naturally infected fish. The stomach and intestine were empty with inflamed gastric walls (Fig. 1, a & b).

### Biochemical identification of bacterial isolates

In the initial bacterial isolation, eighteen bacterial isolates were retrieved; fifteen isolates were from the diseased *S. aurata*, and the other three were from tilapia-trash-feed. *P. putida* grew as white colonies with an opaque center about 1–2 mm in diameter on marine agar. On *Pseudomonas* selective agar, the colonies were more prominent. All isolates were identified biochemically as *P. putida* with 99% probability, the biochemical profile was shown in Table 1.

**Table 1:** Biochemical profile of *P. putida* isolates

Biochemical reactions	Results	Biochemical reactions	Results
Ala-Phe-Pro-Arylamidase	-	Saccharose /Sucrose	-
Adonitol	-	D-Tagatose	-
L- Pyrrolydonyl- Arylamidase	-	D-Trehalose	-
L-Arabitol	-	Sodium Citrate	+
D-Cellobiose	-	Malonate	+
β–Galactosidase	-	5-Keto-D-Gluconate	-
H_2_S production	-	L-Lactate alkalinization	+
β-N-Acetyl –Glucosaminidase	-	α –Glucosidase	-
Glutamyl Arylamidase pNA	-	Succinate Alkalinization	+
D-Glucose	+	β -N-Acetyl –Galactosaminidase	-
γ –Glutamyl –Transferase	+	α –Galactosidase	-
Glucose Fermentation	-	Phosphatase	-
β –Glucosidase	-	Glycine Arylamidase	-
D-Maltose	-	Ornithine Decarboxylase	-
D-Mannitol	-	Lysine Decarboxylase	-
D-Mannose	+	L-Histidine Assimilation	+
β –Xylosidase	-	Courmarate	+
β -alanine arylamidase pNA	-	β –Glucuronidase	-
L-Proline Arylamidase	+	O/129 Resistance (Comp. Vibrio)	+
Lipase	-	Glu-Gly-Arg- Arylamidase	-
Palatinose	-	L-Malate Assimilation	+
Tyrosine Arylamidase	+	Ellman	-
Urease	-	L-Lactate Assimilation	+
D-Sorbitol	-		
Probability		99%	
Number of isolates		18	

### Molecular identification

**Fig. 2:**
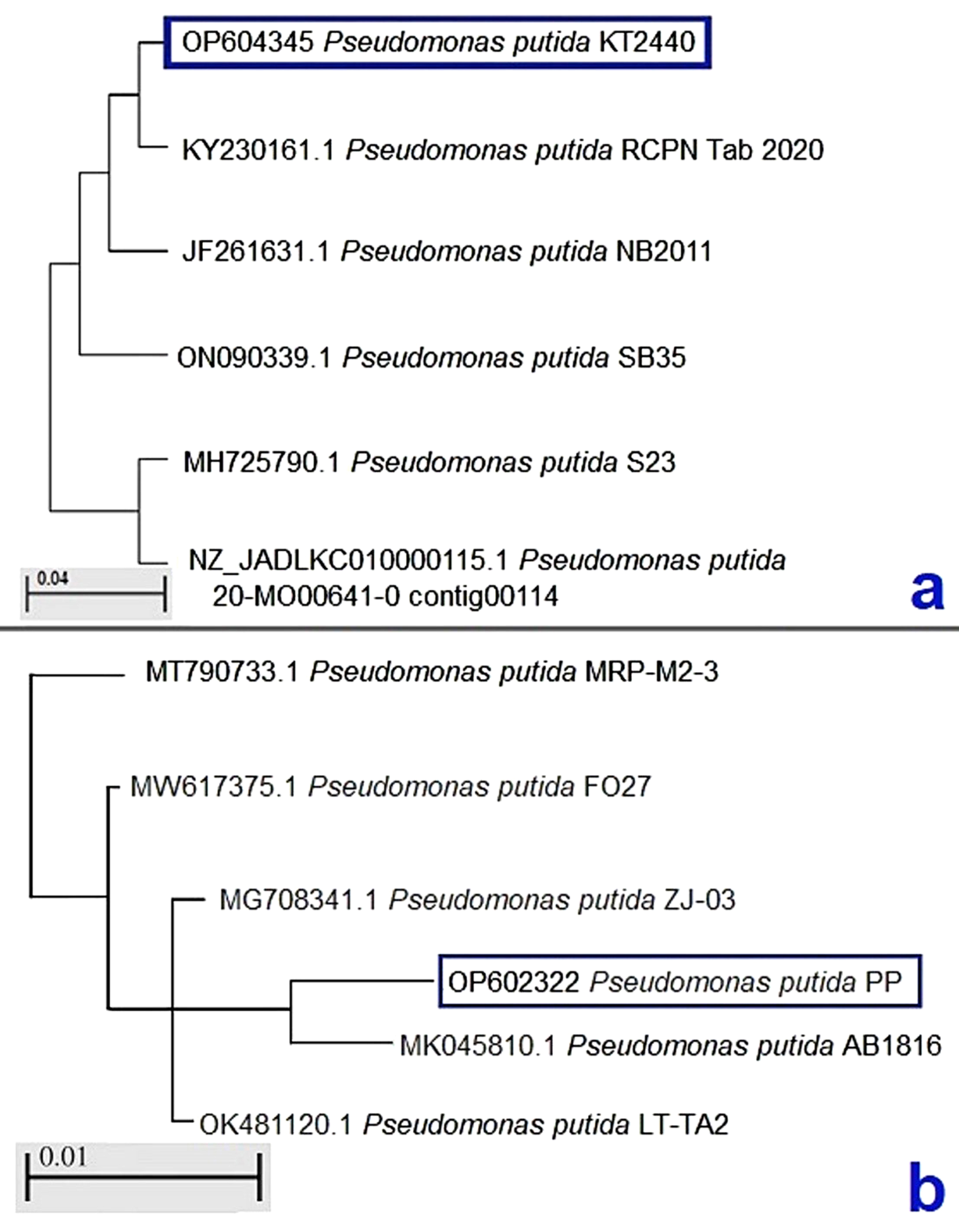
The phylogenetic tree for 16 S-rRNA gene partial sequences for *Pseudomonas putida*. **a**) *P. putida* (KT2440) isolate recovered from diseased *Sparus aurata*. **b**) *P. putida* (PP) isolate retrieved from trash fish (Tilapia)

Bacterial isolates phylogenetically identified as *P. putida* with high similarity to GenBank data. The phylogenetic tree is shown in Fig. 2. The 16 S rRNA gene sequence was deposited in GenBank under the accession numbers (OP604345 and OP602322) for isolate KT 2440 & PP recovered from diseased *S. aurata* and tilapia, respectively. The alignment of both isolate sequences showed 66.3 − 98.1% homology to previously deposited *P. putida* sequences in GenBank. Isolate (KT 2440) retrieved from diseased *S. aurata* was closely related to the following strains: RCPN Table 2020, NB2011, SB35, S23, and 20-MO00641-0 contig00114 isolated from diseased fish in Iran, China, India, India, and Germany, respectively. Regarding isolate (PP) recovered from trash-feed (tilapia), it was closely related to GenBank strains named; LT-TA2 and AB1816 isolated from sturgeon fish and cage-cultured fish. PP isolate was also related to *P. putida* strain FO27 and MRP-M2-3 isolated from water.

### Biofilm production and hemolytic assay

All eighteen *P. putida* isolates can produce biofilm, but only eleven bacterial isolates secreted hemolysin (Fig. S3, a – b, Supplementary data).

### Antibiogram tests

#### Agar disc diffusion test

Tested *P. putida* isolates showed a multidrug resistance profile, as represented in Table 2; Fig. S4a (Supplementary data). All isolates were sensitive to florfenicol, followed by ciprofloxacin (72.22%), then doxycycline (44.4%). All isolates resist ampicillin and sulfamethoxazole-trimethoprim.

**Table 2:** Antibiogram test results for *P. putida* isolates

Antibiotic	SB mm		NSI	NSI %	ZD (mm)	MICB (µg. mL^− 1^)	MIC (µg. mL^− 1^)
Florfenicol	≥ 19		18	100	19–25	≤ 8	0.25–1.0
Ampicillin	≥ 18		0	0	-	-	ND
Erythromycin	≥ 18		2	11.11	18	-	ND
Sulfamethoxazole - Trimethoprim	≥ 19		0	0	0–10.5	-	ND
Ciprofloxacin	≥ 21		13	72.22	22–28	≤ 1	0.0625–1.0
Doxycycline	≥ 16		8	44.44	17–20	-	ND
Tylosin	≥ 18		5	27.77	19–21	-	ND

**SB**: Susceptibility breakpoint zone diameter in mm, **NSI**: Number of susceptible isolates, **NSI%**: Percent of susceptible isolates, **ZD**: Zone diameter range for *P. putida* isolates in mm **MICB**: Minimum inhibitory concentration susceptibility breakpoint in µg.ml^− 1^, **MIC**: Minimum inhibitory concentration ranges of *P. putida* isolates, **ND**: Not determined

#### The broth dilution test

The broth dilution test confirmed the high sensitivity of all *P. putida* isolates to florfenicol with minimum inhibitory concentration ranging between 0.25 and 1 µg. mL ^− 1^. MIC of ciprofloxacin against 13 tested isolates ranged from 0.0625 to 2 µg. mL ^− 1^, as shown in Table 2; Fig. S4b (Supplementary data).

#### The infectivity test

The calculated LD_50_ of *P. putida* for *S. aurata* fingerlings was 6.3 × 10^7^ CFU fish^− 1^, the cumulative mortality at the 7th day post-infection is represented in Table 3.

**Table 3:** Experimental design and mortality rate in *S. aurata* fingerlings IP challenged with *P. putida* isolated from tilapia-trach-feed. (mean ± SE, *n* = 3)

Groups	Inoculum concentration (CFU/Fish)	Fish No./ group	Dead fish No.	Fish mortality (%)
1 (Control)	Normal saline	30	0	0.0 ± 0.00 c
2	5 × 10^6^	30	2	6.7 ± 3.33 bc
3	5 × 10^7^	30	6	20.0 ± 5.77 b
4	5 × 10^8^	30	18	60.0 ± 5.77 a

Control: non-infected non-treated

Means in the same column having different letters are significantly differed at *P* < 0.05.

#### The florfenicol-fish treatment trial

The challenge test indicated the efficacy of florfenicol in protecting *S. aurata* fingerlings against *P. putida* infection. Challenged fish treated with 10 mg kg^− 1^ of florfenicol showed 16.7% mortality, while no mortality was recorded for the group that received 20 mg kg^− 1^, but the non-treated group showed 46.7% mortality after IP bacterial challenge (Table 4).

**Table 4:** Protective effect of florfenicol for *S. aurata* fingerlings IP challenged with 6.3 × 10^7^ CFU fish^− 1^ of *P. putida* isolate. (mean ± SE, *n* = 3)

Groups	Fish No./ group	Florfenicol dose	Dead fish No.	Fish mortality (%)
1	30	10 mg. kg^− 1^	5	16.7 ± 3.33 b
2	30	20 mg. kg^− 1^	0	0.0 ± 0.00 c
3 (+ ve Control)	30	0	14	46.7 ± 3.33 a
4 (-ve Control)	30	0	0	0.0 ± 0.00 c

+ve Control: infected but non-treated; -ve Control: non-infected and non-treated

Means in the same column having different letters are significantly differed at *P* < 0.05

#### Florfenicol level in treated fish serum

Serum florfenicol concentrations for treated fish groups are presented in Table 5. Serum florfenicol levels reached 1.07 and 2.52 µg mL^− 1^ at the 5th day post-drug administration in the group received 10 and 20 mg kg^− 1^ of trash fish, respectively.

**Table 5:** Florfenicol concentrations in serum of *S. aurata* fingerlings at the 5th day post-administration. (mean ± SE, *n* = 3)

Sampling time post-drug administration	Florfenicol concentration in fish serum (µg mL^− 1^)	
	Group 1 (10 mg kg^− 1^ trash fish)	Group 2 (20 mg kg^− 1^ trash fish)
48-h	0.74 ± 0.058 b, x	1.60 ± 0.155 cd, y
72-h	0.84 ± 0.062 ab, x	1.78 ± 0.073 c, y
96-h	0.87 ± 0.057 ab, x	2.21 ± 0.079 b, y
120-h	1.07 ± 0.078 a, x	2.52 ± 0.113 a, y
Two-way ANOVA	*P* value	
Florfenicol (F) level	0.0001	
Sampling time (h)	0.0001	
F level x Sampling time	0.001	

Means having different letters (a – d for columns and x – y for rows) are significantly differed at *P* < 0.05

## Discussion

Trash fish are small fish that do not have commercial value for human consumption; trash fish is accidentally caught from the sea or collected after harvesting the main cultured fish crop (particularly tilapia). Trash fish is mainly used as a natural feed for cultured marine fishes (45–46). Fish farmers preferred to use the trash fish over the formulated fish feed for two reasons; trash fish is more palatable and rabidly consumed than the formulated fish feed. Trash fish has much lower prices (1/15 to 1/20 of formulated marine fish feed price), particularly under the present shortage in grain supply and high prices due to the Russian Ukrainian ware (47–48). Unfortunately, feeding cultured fish on trash fish is associated with many health hazards, including deterioration of farm water quality through the decomposition of uneaten food with ammonia liberation and increasing the microbial load (49–50).

In the present study, *P. putida* was isolated from all tilapia-trash-feed samples used in feeding cultured *S. aurata*. Following our results, many researchers proved the role of trash fish as a source of bacterial and viral fish pathogens. Kim et al. [51] isolated *Streptococcus iniae*, *S. parauberis*, and iridovirus from the trash fish used in cultured flounder feeding. Similarly, Gomez et al. [52] detected red-spotted grouper nervous necrosis virus in 17.22% of trash fish and mollusk samples used for feeding cultured marine fish. Kim [53] concluded that the low-value fish used as feed is a source of *Vibrio harveyi* infection in cultured rockfish, *Sebastes schlegeli.* Nurliyana et al. [54] also reported that *Vibrio sp*. could be transmitted to cultured fish through the newly introduced fish, water, or trash fish. The present study provides additional evidence indicating that feeding cultured fish on trash fish (tilapia) is a possible source of infectious diseases.

In the present study, eighteen *P. putida* were biochemically identified with 99% probability, 15 isolates were retrieved from the clinically diseased fish, and three isolates from tilapia-trash-feed. This pathogen is the leading cause of the present infection affecting *S. aurata*. The phylogenetic analysis of the 16 S rRNA gene sequence for KT 2440 and PP isolates has confirmed the biochemical identification results. The KT 2440 sequence was closely related to *P. putida* isolated from diseased fish.

The virulence test has confirmed the ability of *P. putida* isolate (recovered from tilapia) to infect *S. aurata* fingerlings (satisfy Koch’s postulates), which categorically ensures the responsibility of *P. putida* for this infection. Accordingly, *P. putida* was previously reported from a wide range of diseased freshwater fish, such as rainbow trout and tilapia (17–18 & 20). Infection was recorded in some marine fishes like *D. labrax* and *L. ramada* (19 & 21), but this is the first report documented that *S. aurata* infection with *P. putida* and proved this experimentally.

This report points to the critical role of tilapia-trash-feed as a reservoir for bacterial pathogens that can transmit serious diseases to cultured marine fish fed on diseased or contaminated trash fish. Our results indicated the capability of *P. putida* to withstand high water salinity as it can grow on marine agar containing 19 ppt sodium chloride. He et al. [55]. , indicated the capacity of this pathogen to withstand high water salinity of up to 50 ppt.

*P. putida* isolate was virulent for *S. aurata* fingerlings with LD_50_ equal to 6.3 × 10^7^ CFU Fish^− 1^; this dose was higher than that estimated by Altinok et al. [17] for rainbow trout (5 × 10^6^ CFU Fish^− 1^), this difference could be due to strain pathogenicity, fish species, and rearing conditions among others.

In the present study, all retrieved *P. putida* isolates can produce biofilm, which was in harmony with previous findings [56–58]. Biofilm is one of the most important virulence factors for pathogenic bacteria [59]; however, biofilm-producing bacteria tolerate antibiotics, overcome the innate and adaptive immune response, and resist phagocytosis. Biofilm activates the expression of virulence genes, toxins, and extracellular polymeric substance components [60].

Furthermore, 61.11% of the regained *P. putida* isolates can secrete hemolysin and induce hemolysis for *S. aurata* RBCs. Hemolysin is a potent pore-forming toxin that affects the cytoskeleton and metabolism of many cells like epithelial cells, endothelial cells, erythrocytes, monocytes, and keratinocytes. Hemolysin induces pore formation in the cell membrane, allowing the free flow and leakage of intracellular contents such as electrolytes, proteins, and sugars (61–62). Hemolysin affects host defense through its leucocytolytic activity. Hemolysin damages the host tissues by direct pore formation action or indirectly by stimulating the inflammatory mediators, signal pathways, and iron scavenging [63].

Skin hemorrhages, hyperemia, and congestion of internal organs were the most prominent disease features. Bacterial virulence factors such as hemolysin production and biofilm formation are responsible for disease signs and lesions. Many researchers [18–20], , have described nearly similar disease characteristics in many fish species infected with *P. putida*. Virulence factors are the key to understanding bacterial pathogenesis in fishes; they are responsible for the clinical features and disease severity [9, 26].

In the present study *P. putida* expressed multi-drug resistance profile, it was resistant to ampicillin, erythromycin and tylosin. Multidrug resistance has increased globally, representing a significant public health threat. Multidrug resistant bacterial pathogens from animal sources are considered dangerous for human health, so routine antimicrobial susceptibility testing is essential for identifying the most appropriate antibiotics for the treatment of such infections [67, 68].

All *P. putida* isolates were highly sensitive to florfenicol with MIC equals (0.25–1.0 µg mL^− 1^). Florfenicol is the most recent member of phenicol antibiotic; so, the bacterial resistance is still unfamiliar. Florfenicol is effective against many bacterial pathogens affecting animals and fish; so, it is one of the limited antibiotics approved by the FDA for use in aquaculture [22]. The treatment trial proved the practical efficacy of florfenicol in protecting *S. aurata* fingerlings against *P. putida* infection. This result agrees with previous investigations stating that florfenicol is the most effective treatment for many bacterial infections in aquaculture as streptococcosis, pasteurellosis, vibriosis, and motile *Aeromonas* septicemia [24, 64–66].

In the present study, florfenicol completely protects fish against *P. putida* infection when used at 20 mg. kg^− 1^ trash biomass. Serum florfenicol concentration reached 1.43 and 2.58 µg. mL^− 1^ at 24 and 120 h, respectively, in fish received 20 mg kg^− 1^ trash biomass, which explained drug effectiveness when the serum drug concentration exceeded the MIC of all *P. putida* isolates (0.25–1.0 µg mL^− 1^). Florfenicol at a dose of 10 mg. kg^− 1^ trash biomass effectively lowered the mortality rate to 15%, meanwhile, no mortality was found in fish receiving 20 mg florfenicol per kg of trash biomass. Di Salvo et al. [69] and Abdelhamed et al. [70] reported that florfenicol is effective against bacterial fish diseases when used at 10 mg kg^− 1^ trash biomass and serum drug concentration should exceed MIC.

## Conclusion

*P. putida* is the main cause of disease affecting the studied *S. aurata* fingerlings. It could suggest that contaminated tilapia-trash-feed is the primary source of *P. putida* infection in *S. aurata* fingerlings fed on it. In-feed administration of florfenicol at a dose of 20 mg kg^− 1^ trash biomass effectively protected the challenged *S. aurata* fingerlings against the experimental infection with *P. putida*.

### Electronic supplementary material

Below is the link to the electronic supplementary material.

Supplementary Material 1

## Data Availability

All data will be available upon request.

## References

[1] FAO. (2022): In Brief to The State of World Fisheries and Aquaculture 2022. Towards Blue Transformation. Rome, FAO, 10.4060/cc0463en.

[2] GRFC. (2022): Global Report on Food Crises, 6th Edition, Food Security Information Network (FSIN), https://www.fao.org/3/cb9997en/cb9997en.pdf.

[3] Oliveira PPA, Berndt A, Pedroso AF, Alves TC, Pezzopane JRM, Sakamoto LS, Henrique FL, Rodrigues PHM. Greenhouse gas balance and carbon footprint of pasture-based beef cattle production systems in the tropical region (Atlantic Forest biome). Animal. 2020;14(S3):427–37. 10.1017/S1751731120001822. 10.1017/S1751731120001822 32829724

[4] Teodosio R, Aragao C, Colen R, Carrilho R, Dias J, Engrola S. A nutritional strategy to promote gilthead seabream performance under low temperatures. Aquaculture. 2021;537:736494. 10.1016/j.aquaculture.2021.736494. 10.1016/j.aquaculture.2021.736494

[5] FAO. (2021): Fishery and Aquaculture Statistics, 1976–2019. www.fao.org/fishery/statistics/software/fishstatj/en.

[6] Sadek S. Sea bream culture in Egypt; status, constraints and potential. Fish Physiol Biochem. 2000;22:171–8. 10.1023/A:1007835126731. 10.1023/A:1007835126731

[7] GAFRD. Fish statistics Yearbook 29th edition. General Authority for Fish resources. Egypt: Ministry of Agriculture; 2019.

[8] Khalil MS, Nageeb HM, Aboyadak IM, Ali NG. Treatment of MAS caused by *Aeromonas sobria* in European seabass. Egypt J Aquat Biology Fisheries. 2022;26(5):535–56. https://ejabf.journals.ekb.eg/article_264476.html. 10.21608/ejabf.2022.264476

[9] Toranzo AE, Barreiro S, Casal JF, Figueras A, Magarinos B, Barja JL. Pasteurellosis in cultured gilthead seabream (*Sparus aurata*): first report in Spain. Aquaculture. 1991;99(1–2):1–15. 10.1016/0044-8486(91)90284-E. 10.1016/0044-8486(91)90284-E

[10] Abdel-Aziz M, Eissa A, Hanna M, Abou Okada M. Identifying some pathogenic Vibrio/Photobacterium species during mass mortalities of cultured gilthead seabream (*Sparus aurata*) and European seabass (*Dicentrarchus labrax*) from some Egyptian coastal provinces. Int J Veterinary Sci Med. 2013;1(2):87–95. 10.1016/j.ijvsm.2013.10.004. 10.1016/j.ijvsm.2013.10.004

[11] Aamri FE, Caballero MJ, Real F, Acosta F, Deniz S, Roman L, Padilla D. *Streptococcus iniae* in gilthead seabream (*Sparus aurata, L*.) and red porgy (*Pagrus pagrus, L*): ultrastructural analysis. Vet Pathol. 2015;52(1):209–12. 10.1177/0300985814520638. 24496225 10.1177/0300985814520638

[12] Balebona MC, Andreu MJ, Bordas MA, Zorrilla I, Morinigo MA, Borrego JJ. Pathogenicity of *Vibrio alginolyticus* for cultured gilt-head sea bream (*Sparus aurata L*). Appl Environ Microbiol. 1998;64(11):4269–75. 10.1128/AEM.64.11.4269-4275.1998. 9797276 10.1128/AEM.64.11.4269-4275.1998 PMC106638

[13] Canak O, Akayli T. Bacteria recovered from cultured Gilt-Head Seabream (*Sparus aurata*) and their antimicrobial susceptibilities. Eur J Biology. 2018;77(1):11–7. 10.26650/EuroJBiol.2018.346175. 10.26650/EuroJBiol.2018.346175

[14] Muniesa A, Basurco B, Aguilera C, Furones D, Reverte C, Sanjuan-Vilaplana A, Jansen MD, Brun E, Tavornpanich S. Mapping the knowledge of the main diseases affecting sea bass and sea bream in Mediterranean. Transbound Emerg Dis. 2020;67(3):1089–100. 10.1111/tbed.13482. 31960605 10.1111/tbed.13482

[15] Lalucat J, Gomila M, Mulet M, Zaruma A, Garcia-Valdes E. Past, present and future of the boundaries of the *Pseudomonas* genus: proposal of *Stutzerimonas* gen. Nov. Syst Appl Microbiol. 2021;45(1):126289. 10.1016/j.syapm.2021.126289. 34920232 10.1016/j.syapm.2021.126289

[16] Altinok I, Kayis S, Erol Capkin E. *Pseudomonas putida* infection in rainbow trout. Aquaculture. 2006;261(3):850–5. 10.1016/j.aquaculture.2006.09.009. 10.1016/j.aquaculture.2006.09.009

[17] Eissa NME, El-Ghiet A, Shaheen EN A. A. and, Abbass A. Characterization of Pseudomonas species isolated from Tilapia *Oreochromis Niloticus* in Qaroun and Wadi-El-Rayan Lakes, Egypt. Global Vet. 2010;5(2):116–21. 10.13140/2.1.5002.4961. 10.13140/2.1.5002.4961

[18] El-Barbary M, Hal A. Isolation and molecular characterization of some bacterial pathogens in El-Serw fish farm, Egypt. Egypt J Aquat Biology Fisheries. 2016;20(4):115–27. 10.21608/EJABF.2016.11183. 10.21608/EJABF.2016.11183

[19] Oh WT, Kim JH, Jun JW, Giri SS, Yun S, Kim HJ, Kim SG, Kim SW, Han SJ, Kwon J, Park SC. Genetic characterization and pathological analysis of a Novel Bacterial Pathogen, *Pseudomonas Tructae*, in Rainbow Trout (*Oncorhynchus mykiss*). Microorganisms. 2019;7(10):432. 10.3390/microorganisms7100432. 31658660 10.3390/microorganisms7100432 PMC6843698

[20] Urku C. Isolation and characterization of Pseudomonas putida caused granulomas in cultured sea bass (*Dicentrarchus labrax*) in Turkey. J Hellenic Veterinary Med Soc. 2021;72(1):2661–8. 10.12681/jhvms.26748. 10.12681/jhvms.26748

[21] Zeng Q, Liao C, Terhune J, Wang B. Impacts of florfenicol on the microbiota landscape and resistome as revealed by metagenomic analysis. Microbiome. 2019;7:155. 10.1186/s40168-019-0773-8. 31818316 10.1186/s40168-019-0773-8 PMC6902485

[22] Barreto FM, da Silva MR, Braga PAC, Bragotto APA, Hisano H, Reyes FGR. Evaluation of the leaching of florfenicol from coated medicated fish feed into water. Environnemental Pollution. 2018;242(Part B):1245–52. 10.1016/j.envpol.2018.08.017. 10.1016/j.envpol.2018.08.017 30118912

[23] Aboyadak IM, Ali NG, Abdel-Aziz MM, Gado MS, El-Shazly KA. Role of some antibacterial drugs in control *Streptococcus iniae* infection in *Oreochromis niloticus*. J Pharmacol Clin Res. 2016;1:555573. 10.19080/JPCR.2016.01.555573. 10.19080/JPCR.2016.01.555573

[24] Austin B, Austin DA. Bacterial fish pathogens: disease of farmed and wild fish. 6th ed. Switzerland: Springer International Publishing; 2016. 10.1007/978-3-319-32674-0.

[25] El-Bahar HM, Ali NG, Aboyadak IM, Khalil SA, Ibrahim MS. Virulence genes contributing to *Aeromonas hydrophila* pathogenicity in *Oreochromis niloticus*. Int Microbiol. 2019;22:479–90. 10.1007/s10123-019-00075-3. 30989358 10.1007/s10123-019-00075-3

[26] Ali NG, Ali TE, Aboyadak IM, Elbakry MA. Controlling *Pseudomonas aeruginosa* infection in *Oreochromis niloticus* spawners by cefotaxime sodium. Aquaculture. 2021a;544:737107. 10.1016/j.aquaculture.2021.737107. 10.1016/j.aquaculture.2021.737107

[27] Ali NG, Ali TE, Kamel MF, Saleh R, Sherif AH, Aboyadak IM. Eradication of *Livoneca redmanii* infestation in cultured *Argyrosomus regius*. Aquaculture. 2022;558:738373. 10.1016/j.aquaculture.2022.738373. 10.1016/j.aquaculture.2022.738373

[28] Fujiyoshi S, Muto-Fujita A, Maruyama F. Evaluation of PCR conditions for characterizing bacterial communities with full-length 16S rRNA genes using a portable nanopore sequencer. Sci Rep. 2020;10:12580. 10.1038/s41598-020-69450-9. 32724214 10.1038/s41598-020-69450-9 PMC7387495

[29] Lee PY, Costumbrado J, Hsu CY, Kim YH. Agarose gel electrophoresis for the separation of DNA fragments. J Visual Experiments. 2012;62(1–5):e3923. 10.3791/3923. 10.3791/3923 PMC4846332 22546956

[30] Bal H, Hujol J. Introduction to Basic Local Alignment Search Tool. Java for Bioinformatics and Biomedical Applications. Boston, MA: Springer; 2007. 10.1007/978-0-387-37237-2_2.

[31] Kumar S, Stecher G, Tamura K. MEGA7: molecular evolutionary genetics analysis version 7.0 for bigger datasets. Mol Biol Evol. 2016;33(7):1870–4. 10.1093/molbev/msw054. 27004904 10.1093/molbev/msw054 PMC8210823

[32] Evans BC, Nelson CE, Yu SS, Beavers KR, Kim AJ, Li H, Nelson HM, Giorgio TD, Duvall CL. Ex vivo red blood cell hemolysis assay for the evaluation of pH-responsive endosomolytic agents for cytosolic delivery of biomacromolecular drugs. J Visualized Experiments. 2013;73e50166. 10.3791/50166. 10.3791/50166 PMC3626231 23524982

[33] Corte L, Pierantoni DC, Tascini C, Roscini L, Gianluigi Cardinali C. Biofilm Specific activity: a measure to quantify Microbial Biofilm. Microorganisms. 2019;7:73. 10.3390/microorganisms7030073. 30866438 10.3390/microorganisms7030073 PMC6463164

[34] Ali NG, El-Nokrashy AM, Gouda MY, Aboyadak IM. Summer mortality syndrome affecting cultured European Seabass at Kafrelsheikh Province, Egypt. Front Mar Sci. 2021;8:717360. 10.3389/fmars.2021.717360. 10.3389/fmars.2021.717360

[35] CLSI. (2016). Clinical and Laboratory Standards Institute, Document M45-A. Methods for antimicrobial dilution and disk susceptibility of infrequently isolated or fastidious bacteria; approved guideline. Pennsylvania, USA.

[36] Ali NGM, Aboyadak IM, El-Sayed HS. Chemotherapeutic control of Gram-positive infection in white sea bream (Diplodus sargus, Linnaeus 1758) broodstock. Veterinary World. 2019;12(2):316–24. 10.14202/vetworld.2019.316-324. 31040576 10.14202/vetworld.2019.316-324 PMC6460867

[37] Fang D, Mei J, Xie J, Qiu W. The effects of Transport Stress (temperature and vibration) on blood biochemical parameters, oxidative stress, and Gill Histomorphology of Pearl Gentian groupers. Fishes. 2023;8(4):218. 10.3390/fishes8040218. 10.3390/fishes8040218

[38] Saleh NE, Helal M, Ali NG, Abbas E, Abdel-Tawwab M. Effects of using vital wheat gluten in practical diets on growth, intestinal histopathology, proinflammation related gene expression, and resistance of white seabream (*Diplodus sargus*) to *Staphylococcus epidermidis* infection. Aquaculture. 2021;537:736508. 10.1016/j.aquaculture.2021.736508. 10.1016/j.aquaculture.2021.736508

[39] Reed LJ, Muench H. A simple method of estimating 50% endpoints. Am J Epidemiol. 1938;27(3):493–7. 10.1093/oxfordjournals.aje.a118408. 10.1093/oxfordjournals.aje.a118408

[40] Boyd CE. Water quality in warm water fishponds. Auburn, AL, USA: Auburn University Agriculture Experimental Station; 1984.

[41] Boyd CE, Tucker CS. (2012): Pond Aquaculture Water Quality Management. Springer New York, NY, ISBN: 978-1-4613-7469-5, 10.1007/978-1-4615-5407-3.

[42] Yang J, Sun G, Qian M, Huang L, Ke X, Yang B. Development of a high-performance liquid chromatography method for the determination of florfenicol in animal feedstuffs. J Chromatogr B. 2017;1068–1069:9–14. 10.1016/j.jchromb.2017.09.045. 10.1016/j.jchromb.2017.09.045 29028619

[43] Dytham C. Choosing and using statistics: a biologist’s guide. London, UK: Blackwell Science Ltd.; 2011.

[44] Hecht T, Jones CLW. (2009): Use of wild fish and other aquatic organisms as feed in aquaculture – a review of practices and implications in Africa and the Near East. *In* M.R. Hasan and M. Halwart, editors. Fish as feed inputs for aquaculture: practices, sustainability and implications. FAO Fisheries and Aquaculture Technical Paper. No. 518. Rome, FAO. pp. 129–157.

[45] Hasan MR. (2012): Transition from low-value fish to compound feeds in marine cage farming in Asia. FAO Fisheries and Aquaculture Technical Paper. No. 573. Rome, FAO. 2012. 198 pp, https://www.fao.org/apfic/publications/detail/en/c/419427/.

[46] Ben Hassen T, El Bilali H. Impacts of the Russia-Ukraine War on Global Food Security: towards more sustainable and resilient Food systems? Foods. 2022;11(2301). 10.3390/foods11152301. 10.3390/foods11152301 PMC9368568 35954068

[47] Jagtap S, Trollman H, Trollman F, Garcia-Garcia G, Parra-López C, Duong L, Martindale W, Munekata PES, Lorenzo JM, Hdaifeh A, Hassoun A, Salonitis K, Afy-Shararah M. The Russia-Ukraine conflict: its implications for the global food supply chains. Foods. 2022;11(2098). 10.3390/foods11142098. 10.3390/foods11142098 PMC9318935 35885340

[48] Allsopp M, Johnston P, Santillo D. (2008): Greenpeace Research Laboratories Technical Note 01/2008. Amsterdam, The Netherlands, https://www.greenpeace.to/greenpeace/wp-content/uploads/2011/05/Aquaculture_Report_Technical.pdf.

[49] Mozumder MMH, Uddin MM, Schneider P, Raiyan MHI, Trisha MGA, Tahsin TH, Newase S. Sustainable Utilization of Fishery Waste in Bangladesh—A qualitative study for a circular Bioeconomy Initiative. Fishes. 2022;7:84. 10.3390/fishes7020084. 10.3390/fishes7020084

[50] Kim JH, Gomez DK, Choresca CH Jr, Park SC. Detection of major bacterial and viral pathogens in trash fish used to feed cultured flounder in Korea. Aquaculture. 2007;22(1–4):105–10. 10.1016/j.aquaculture.2007.09.008. 10.1016/j.aquaculture.2007.09.008

[51] Gomez DK, Mori K-I, Okinaka Y, Nakai T, Park SC. Trash fish can be a source of betanodaviruses for cultured marine fish. Aquaculture. 2010;302:158–63. 10.1016/j.aquaculture.2010.02.033. 10.1016/j.aquaculture.2010.02.033

[52] Kim D-H. Low-value fish used as feed is a source of Disease in Farmed Fish. Fishers Aquat Sci. 2015;18(2):203–9. 10.5657/FAS.2015.0203. 10.5657/FAS.2015.0203

[53] Nurliyana M, Amal M, Zamri-Saad M, Ina-Salwany M. Possible transmission routes of *Vibrio* spp. in tropical cage-cultured marine fishes. Lett Appl Microbiol. 2019;68:485–96. 10.1111/lam.13146. 30834548 10.1111/lam.13146

[54] He Y, Wu Z, Wang, Ye B-C, Zhang F, Liu X. Different responses of *Capsicum annuum* L. Root and shoot to salt stress with *Pseudomonas putida* Rs-198 inoculation. J Plant Growth Regul. 2019;38:799–811. 10.1007/s00344-018-9891-y. 10.1007/s00344-018-9891-y

[55] Fernandez M, Porcel M, de la Torre J, Molina-Henares MA, Daddaoua A, Llamas MA, Roca A, Carriel V, Garzon I, Ramos JL, Alaminos M, Duque E. Analysis of the pathogenic potential of nosocomial *Pseudomonas putida* strains. Front Microbiol. 2015;25:6:871. 10.3389/fmicb.2015.00871. 10.3389/fmicb.2015.00871 PMC4548156 26379646

[56] Molina L, Udaondo Z, Duque E, Fernandez M, Bernal P, Roca A, de la Torre J, Ramos JL. Specific Gene Loci of clinical *Pseudomonas putida* isolates. PLoS ONE. 2016;28(11):e0147478. 10.1371/journal.pone.0147478. 10.1371/journal.pone.0147478 PMC4731212 26820467

[57] Ouyang K, Mortimer M, Holden PA, Cai P, Wu Y, Gao C, Huang Q. Towards a better understanding of Pseudomonas putida biofilm formation in the presence of ZnO nanoparticles (NPs): role of NP concentration. Environ Int. 2020;137:105485. 10.1016/j.envint.2020.105485. 32004708 10.1016/j.envint.2020.105485

[58] Sedarat Z, Taylor-Robinson AW. Biofilm formation by pathogenic Bacteria: applying a *Staphylococcus aureus* Model to appraise potential targets for therapeutic intervention. Volume 11. Pathogens; 2022. p. 388. 10.3390/pathogens11040388. 10.3390/pathogens11040388 PMC9027693 35456063

[59] Mann R, Holmes A, McNeilly O. Evolution of biofilm-forming pathogenic bacteria in the presence of nanoparticles and antibiotic: adaptation phenomena and cross-resistance. J Nanobiotechnol. 2021;19:291. 10.1186/s12951-021-01027-8. Cavaliere, R.Sotiriou, G. A., Rice, S. A. and Gunawan, C. 10.1186/s12951-021-01027-8 PMC8474960 34579731

[60] Elliott SJ, Srinivas S, Albert MJ, Alam K, Robins-Browne RM, Gunzburg ST, Mee BJ, Chang BJ. Characterization of the roles of hemolysin and other toxins in enteropathy caused by alpha-hemolytic Escherichia coli linked to human diarrhea. Infect Immun. 1998;66(5):2040–51. 10.1128/iai.66.5.2040-2051.1998. 9573087 10.1128/iai.66.5.2040-2051.1998 PMC108161

[61] Divyakolu S, Chikkala R, Ratnakar KS, Sritharan V. Hemolysins of *Staphylococcus aureus* - An Update on their Biology, Role in Pathogenesis and as targets for Anti-virulence Therapy. Adv Infect Dis. 2019;9(2):80–104. 10.4236/aid.2019.92007. 10.4236/aid.2019.92007

[62] Welch RA. Pore-forming cytolysins of gram-negative bacteria. Mol Microbiol. 1991;5(3):521–8. 10.1111/j.1365-2958.1991.tb00723.x. 2046545 10.1111/j.1365-2958.1991.tb00723.x

[63] Algammal AM, Alfifi KJ, Mabrok M, Alatawy M, Abdel-moneam DA, Alghamdi S, Azab MM, Ibrahim RA, Hetta HF, El-Tarabili RM. Newly emerging MDR B. cereus in Mugil Seheli as the First Report Commonly Harbor nhe, hbl, cytK, and pc-Plc virulence genes and bla1, bla2, tetA, and ermA Resistance genes. Infect Drug Resist. 2022;15(2167–2185). 10.2147/IDR.S365254. 10.2147/IDR.S365254 PMC9052338 35498633

[64] Algammal AM, Mabrok M, Ezzat M, Alfifi KJ, Esawy AM, Elmasry N, El-Tarabili RM. Prevalence, antimicrobial resistance (AMR) pattern, virulence determinant and AMR genes of emerging multi-drug resistant *Edwardsiella tarda* in Nile tilapia and African catfish. Aquaculture. 2022;548:737643. 10.1016/j.aquaculture.2021.737643. 10.1016/j.aquaculture.2021.737643

[65] Gaunt PS, Gao D, Sun F, Endris R. Efficacy of florfenicol for control of mortality caused *by Flavobacterium columnare* infection in channel catfish. J Aquat Anim Health. 2010;22:115–22. 10.1577/H09-057.1. 20848886 10.1577/H09-057.1

[66] Kogiannou D, Nikoloudaki C, Katharios P, Triga A, Rigos G. Evaluation and depletion of florfenicol in European seabass Dicentrachus Labrax. Veterinary Med Sci. 2020;7(3):987–97. 10.1002/vms3.415. 10.1002/vms3.415 PMC8136953 33369159

[67] Zhai Q, Chang Z, Li J, Li J. Effect of combined florfenicol and chlorogenic acid to treat acute hepatopancreatic necrosis disease in *Litopenaeus vannamei* caused by *Vibrio parahaemolyticus*. Aquaculture. 2021;547:737462. 10.1016/j.aquaculture.2021.737462. 10.1016/j.aquaculture.2021.737462

[68] Di Salvo A, Rocca GD, Terzetti E, Malvisi J. Florfenicol depletion in edible tissue of rainbow trout, *Oncorhynchus mykiss* (Walbaum), and sea bream, *Sparus aurata L*. J Fish Dis. 2013;36:685–93. 10.1111/j.1365-2761.2012.01437.x. 23384074 10.1111/j.1365-2761.2012.01437.x

[69] Abdelhamed H, Ozdemir O, Waldbieser G, Perkins AD, Lawrence ML, Karsi A. Effects of florfenicol feeding on diversity and composition of the intestinal microbiota of channel catfish (*Ictalurus punctatus*). Aquac Res. 2019;50:3663–72. 10.1111/are.14325. 10.1111/are.14325

[70] CCAC. (2005): Guidelines on the care and use of fish in research, teaching and testing. Can Council Anim Care, 1510–130 Albert Street Ottawa on Canada, K1P 5G4, ISBN: 0-919087-43–4.

[71] NACLAR. (2004): National Advisory Committee for Laboratory Animals Research. 20 Biopolis way #08 – 01 Centros Singapore 138668, http://research.ntu.edu.sg/guides/Documents/Ethics/NACLAR-guide%20Lines.pdf.

