# Supplementary material for: Identification and florfenicol-treatment of pseudomonas putida infection in gilthead seabream (Sparus aurata) fed on tilapia-trash-feed
Source: BMC Vet Res. 2024 Apr 25;20:156. doi: 10.1186/s12917-024-04004-z (PMC11044311; doi:10.1186/s12917-024-04004-z)
Supplement: Supplementary file 1 — Supplementary Material 1 [file 12917_2024_4004_MOESM1_ESM.docx]

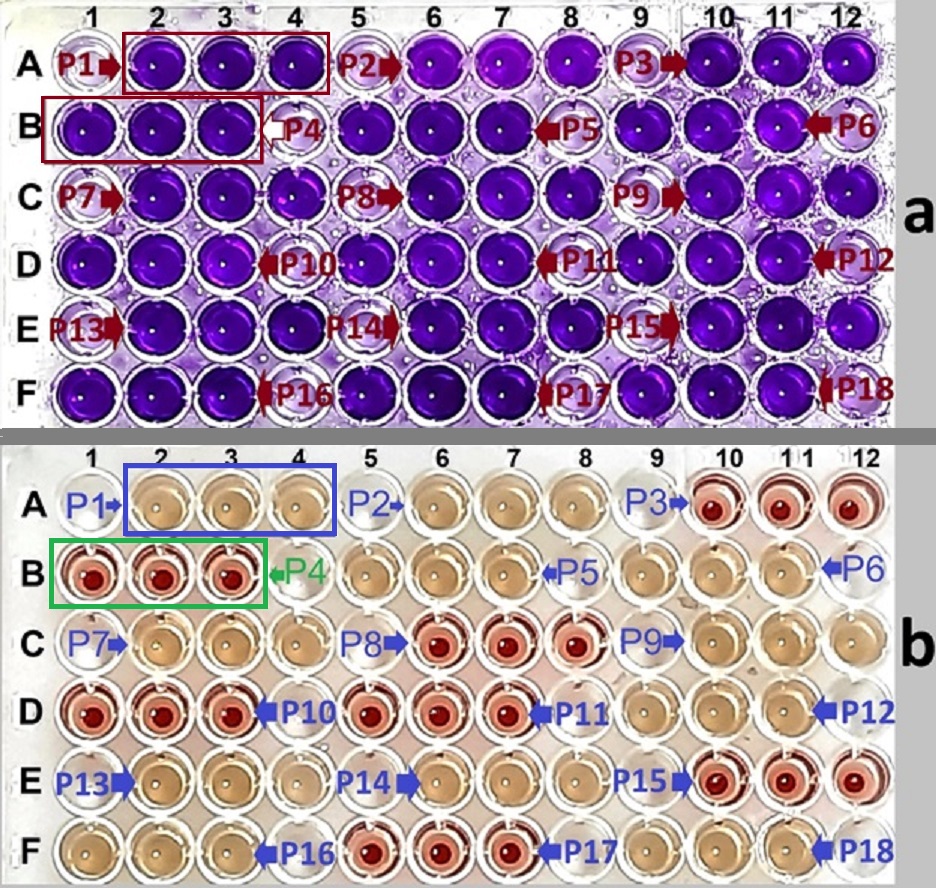


**Figure 3: a)**Biofilm production assay indicating that all tested P. putida isolates can produce biofilm. **b)** Hemolytic assay showed the ability of P. putida isolates number 1, 2, 5, 6, 7, 9, 12, 13, 14, 16 and 18 to produce hemolysin and hemolyze RBCs. The blue rectangle showed positive hemolysis, and the green rectangle showed a negative result as button shape RBCs collection in the bottom of the well. Each test was performed in triplicates. Arrow pointed to the three wells for each isolate.


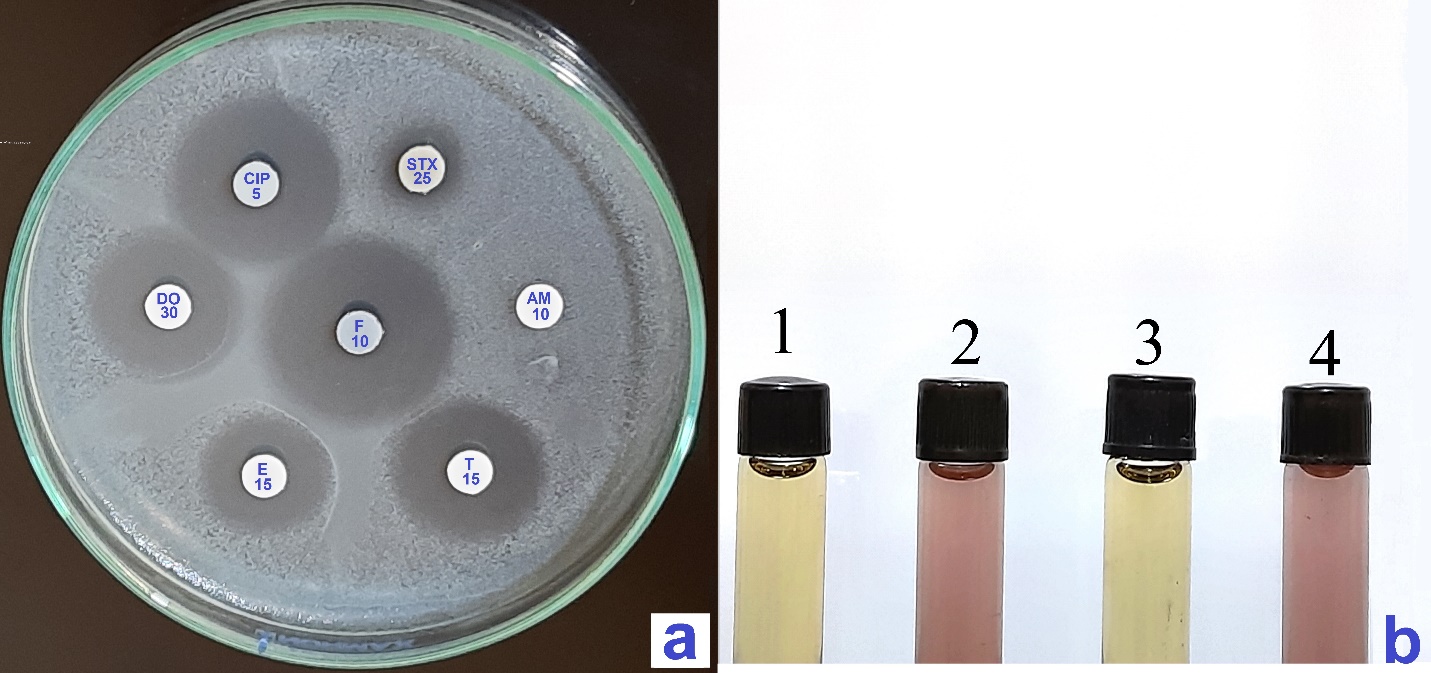


**Figure 4: a)** *P. putida* isolate showed high susceptibility to florfenicol, ciprofloxacin, tylosin, doxycycline and erythromycin. **b)** MIC of florfenicol and ciprofloxacin for *P. putida* (tubes 1 and 2 contain florfenicol 0.25 and 0.125 µg mL ^-1^ and tubes 3 and 4 contain ciprofloxacin 1 & 0. 5 µg mL ^-1^), respectively.
